# Introduced substrates trigger colonization by reef-associated fish in a degraded coastal system

**DOI:** 10.1371/journal.pone.0317431

**Published:** 2025-01-30

**Authors:** Maryann S. Watson, Jon Dickson, Oscar Franken, Laura L. Govers, Tjisse van der Heide, Sterre Witte, Britas Klemens Eriksson

**Affiliations:** 1 Groningen Institute for Evolutionary Life Sciences, University of Groningen, Groningen, Netherlands; 2 Royal Netherlands Institute for Sea Research (NIOZ), Coastal Systems, Den Hoorn, Noord Holland, Netherlands; MARE – Marine and Environmental Sciences Centre, PORTUGAL

## Abstract

Coastal reefs benefit the survival and growth of mobile organisms by providing shelter and increased food availability. Under increasing pressure from human activities, the coverage of subtidal reefs has decreased along the world’s coasts. This decline is motivating efforts to restore these important habitats by re-introducing hard substrates into the coastal zone. However, many such projects use artificial substrates, such as concrete or metal, that are not naturally occurring in the marine environment. We experimentally introduced hard substrates that were either historically common in a soft sediment-dominated ecosystem, or are mimicking these substrates with biodegradable material, and monitored the substrates for mobile species use (fish and invertebrates). Six substrates were tested: cockle shells, rocks of two sizes (cobbles and pebbles), wood, artificial reefs of calcium carbonate with shell fragments, and biodegradable structures based on potato starch. Within one year, fish and prawns were already attracted to all of the introduced substrates. On average, fish were nearly five times as abundant and prawn abundance increased nearly 30-fold on the artificial reefs, compared to the bare sand bottom control. The community composition on the reefs differed significantly from the sand bottom community, but there were no differences between the types of introduced substrates. Interestingly, the substrates attracted reef-associated fish, but also soft-sediment dependent species, such as different species of flatfish and gobies. Our results show that, even over shorter timespans, introductions of hard substrates provide opportunities to support associated mobile communities in degraded soft-sediment systems.

## Introduction

Coastal marine ecosystems are dynamic and complex seascapes of diverse habitat types supporting high biodiversity and abundances of species. Reefs of both biogenic and geogenic origins are important contributors to habitat heterogeneity in these systems, and provide the foundations for associated sessile and mobile communities [1, 2]. Long-term human alteration and engineering of the coast, exposure to pollution, and targeted commercial exploitation has led to widespread degradation of habitat-forming structures, such as seagrass beds, mangrove forests, and both geological and biogenic reefs [2–9]. This has resulted in a loss of the important functions these coastal habitats provide, including essential feeding and nursery areas, and as stopover habitat for migrating species [10–12]. This habitat loss has also been paralleled by declines and collapse of many coastal fish populations caused by targeted commercial fisheries [13–15].

In efforts to restore complex habitat and support fish communities, introductions of hard substrates such as artificial reefs have been performed with varying success, and with varying acceptance in the scientific community [16–19]. Experimental introductions of artificial reefs and substrates is a key step in understanding the efficacy of different types of structures, materials and locations within specific environments before initiating large-scale restoration projects [19, 20].

Restoration of fish habitat through the introduction of substrates and structure has long been implemented as a strategy to support declining fish communities [7, 21–23]. Adding hard substrates, usually bundles of wood or rocks, has been used by artisanal fisheries for hundreds of years to increase fisheries yields [24, 25], and more recently introductions of diverse artificial reef structures have become a common management strategy [26].

Many successful artificial reef introductions and rocky reef restorations have demonstrated increased habitat use by associated fish species [e.g. 7, 22, 27, 28]. Although numbers of artificial reef sites and studies of their effects are growing globally, these are primarily within tropical habitats (e.g. mangroves, coral reefs) with fewer examples within temperate regions [29]. Yet, studies from temperate oyster reefs show that restoration of habitat complexity promotes fish community recovery similar to what is observed in tropical systems [30, 31].

The Wadden Sea is a vast temperate tidal soft-sediment ecosystem, composed of intertidal flats and subtidal banks and gullies, and is a highly productive coastal environment [32]. Multiple habitat types are found within the Wadden Sea, though the abundance and distribution of these have changed over time. These include biogenic habitats of mussel (*Mytilus edulis*) and oyster (historically *Ostrea edulis*, but now dominated by the introduced *Crassostrea gigas)* beds. Historically, it was connected to a large inland marsh landscape and received terrestrial inputs of driftwood and hardened peat, and rocks of different sizes (from glacial deposits of boulders to gravel). The benthic habitats of the Wadden Sea have been impacted by bottom-contact fisheries, including direct harvest of the hard substrates themselves [13, 33, 34], as well as coastal development and dredging practices homogenizing the sea floor [2]. For example, in nearby waters the stone fishing industry has removed millions of boulders amounting to areas of at least tens of km^2^ of hard substrates [27, 35]. In the Wadden Sea marine aggregate extraction is still active and historically exceeded 4.5 million m^3^ of sand and 140 000 m^3^ of shellfish material every year [36]. Additionally, the supply of woody debris from inland sources have been stopped by dykes and other coastal hardening (e.g. sea walls and dykes) fringing almost the entirety of the Wadden Sea coastline. In addition, biological sublittoral reef structures such as European oysters (*Ostrea edulis*), and the polychaete *Sabellaria spinulosa* have functionally disappeared due to bottom trawling [13, 37, 38]. Consequently, the presence of sublittoral hard structures has most likely decreased dramatically in the Wadden Sea compared to historic records.

Here, we examine the fish and mobile species found at a small-scale artificial reef introduction experiment in the Wadden Sea over the period of one year with aim to investigate whether they attract mobile species. We also evaluate what the assemblages of species found could mean for habitat restoration in this ecosystem. To account for the large variety of natural substrates that have declined in the Wadden Sea, we tested the effect of six different types of substrate as artificial reefs on the fish community: cockle shells, large (cobbles) and small stones (pebbles), wood chunks, cobbles mounted onto a concrete base and Biodegradable Elements made of starch (BESE-Elements). We hypothesized that the artificial reef-cages would increase fish abundance and diversity compared to nearby, unaltered regions. Further, we anticipate more effectiveness at artificial substrates that mimic dominate natural substrates in the Wadden sea, such as cockle shells and pebbles, compared to substrates that are naturally less abundant (i.e., wood, cobbles, concrete and BESE structures).

## Methods

### Experimental set-up

**Fig 1:**
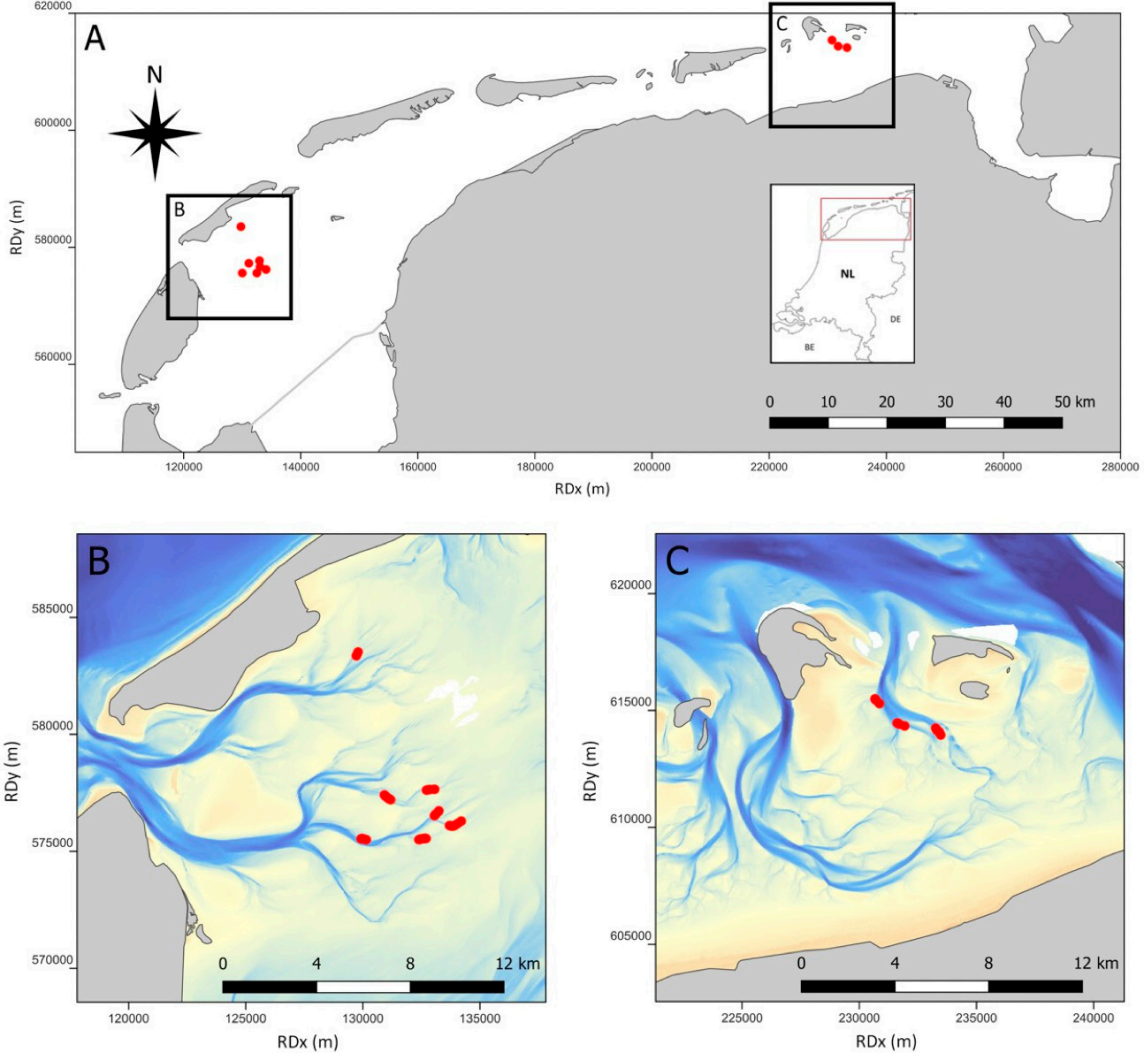
(A) Locations of reef-cage blocks (yellow circles) deployed in May 2020 in the Dutch Wadden Sea. One block consists of six reef-cages each containing a different hard substrate material. Locations of reef-cage blocks (B) in the western Wadden Sea in the Eirlandse Gat tidal basin, and (C) in the eastern Wadden Sea tidal basin of Schild.

**Fig 2:**
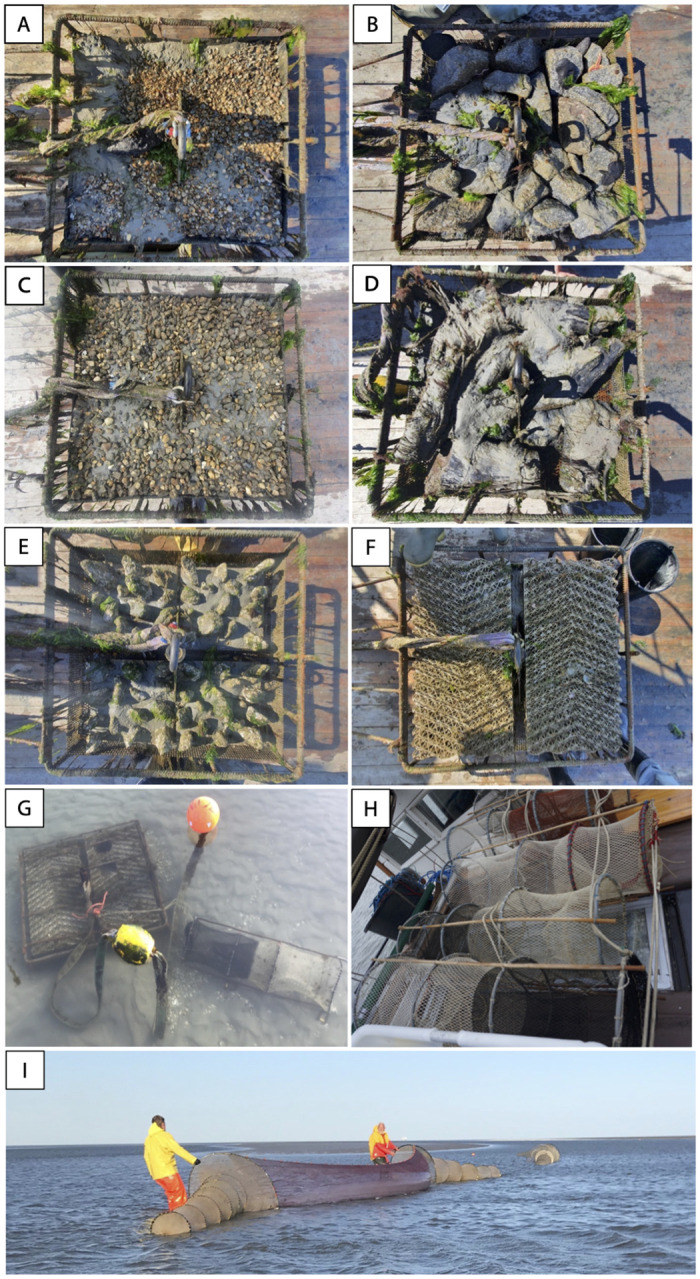
1x1m reef-cages during a lifting event for benthic surveys on different substrates (A) shell, (B) cobble, (C) pebble, (D) wood, (E) “Reef”, and (F) BESE. (G) A fishing trap (‘*kubben*’) attached to a reef-cage containing BESE at low tide, (H) fishing traps, (I) fykes (‘*schietfuiken’*).

The habitat restoration experiment was part of a project to assess the response of benthic and mobile communities to the introduction of different types of hard substrates in the Dutch Wadden Sea. Six different types of substrates were introduced in metal cages at 10 sites across two tidal basins in May 2020 which sit within the Wadden Sea World Heritage Site and Natura 2000 site (Fig 1). The cages were 1m x 1m wide and 20cm deep, with a 10cm long supporting leg at each corner. Each reef-cage contained one of six substrate treatments: empty cockle shells, granite cobbles (6-25cm), pebbles (0.4 -6cm), pieces of bog wood collected from the Wieden National Park, Biodegradable Ecosystem Engineering Elements made of starch (BESE-Elements, BESE Ecosystem Restoration Products, Culemborg, The Netherlands), and silex boulders mounted onto a concrete base, manually coated with a calcareous BESE Reef paste (treatment termed ‘Reef’; Fig 2A–2F). For a detailed explanation of the design of the cages, and the composition and construction of the substratum [see 39]. At each site, a block of six reef-cages—one of each substrate type—were introduced in a tidal channel at depths of between one to three meters. The cages were placed at least 10 meters from each other. The reef-cages were removed in November 2021.

### Fish sampling

#### Trap fishing

We sampled the reef cages for mobile species by fishing at five periods over the experiment deployment (September 2020, April 2021, May/June 2021, August 2021, and September/October 2021). Fishing activities were carried out under Permit PF-2022/245257, issued by the Province of Friesland. Fishing was conducted at least one month following other survey events (detailed below) to avoid disturbance. We used unbaited fish traps with one opening; these traps turn with the strong tidal currents in the area so that the opening is aways on the leeward side (trap: 1m x 25cm x 25cm and with a 15cm net opening, 14mm mesh). This means that fish need to actively swim into the trap. One trap was fastened to the center of each cage so that it moved around close to the reef during the tidal cycle (Fig 2G & 2H), and six reference traps were set on sandy substrates in between and next to the cages in the cage block, at least 5 meters from the nearest cage. Traps and were set for 2 tidal cycles and lifted each tidal cycle (low tide to low tide; ~12 hours) to count and measure catch during daytime and overnight periods. Catch was identified to species and total lengths of fish were measured and returned to the water as quickly as possible (no species were collected, and all fish were handled and released by a certified animal handler (M.S.W)). In the case of juvenile flatfish of the species European plaice (*Pleuronectes platessa*), and European flounder (*Platichthys flesus*), these were grouped into Flatfish spp. due to difficulty of identification of these species at the juvenile stage.

Across all sampling, a total of 222 traps were set on reef cages and 251 reference traps on the sand between the reefs. Numbers differed slightly between some sets as a result of reef cages becoming buried due to mobile sediment or losing substrate to wave action and currents. Not all blocks could be sampled at the same frequencies due to logistics and weather restrictions.

#### Fyke fishing

To sample the wider mobile community in the areas of the reef-cage blocks, two large fyke nets (1m tall at the entrances, and two 8m long nets, mesh size 12mm, connected by a 7m sheet of netting; Fig 2I) were placed in the tidal channels on sand bottom alongside the blocks at the same time that traps were set at the reef-cages and no-reef controls. These were also lifted twice at 12-hour intervals to count and measure catches as above.

#### Cage lifting

Cages were lifted seasonally onto a ship deck for sessile community surveys and then replaced in the same locations (August 2020, October 2020, March 2021, August 2021, and November 2021). During these lifts, the number and length of fish species found on the top layer of the substrate, or that fell out of the cage when lifted onto the ship were recorded. Since the reef structures will trap mobile species differently during lifts, a statistical comparison of catches would be heavily biased. Therefore, these data represented in (S1.1 Fig in S1 File) for reference only.

### Analysis

We examined catch abundances and fish species diversity using a catch (or species) per unit effort (CPUE) in each trap or fyke net per 12 hours of fishing. The catch from the traps were used for statistical analyses testing effects of the reef-cages, while the catch from the larger fyke nets were only used to show differences visually. There was a high number of empty traps throughout, which is challenging to handle with multivariate analyses [40]. In addition the catch was dominated by one single species. Therefore, we mainly analyzed the effects of the cage treatments using univariate analyses, which we then complemented with a multivariate analyses where we removed all the empty traps from the data [41].

First, the effect of the presence of an artificial reef (2 levels: reef-cage vs no-reef cage) and the effect of having different reef substrates in the reef cages (6 levels: cockle shells vs cobbles vs pebbles vs wood vs concrete vs BESE structures) on the catch of mobile species in the traps, were analyzed separately using Generalized linear mixed models (GLMM) with Penalized Quasi-Likelihood [nlme package in R; 42, 43]. Due to the high number of empty traps, quasi-poisson distributions were used to account for overdispersion.

GLMM’s were constructed for each of the response variables: total fish abundance, number of fish species, numbers of prawn, and numbers of crabs per trap at each reef-cage. In the model comparisons we included one of the two cage treatments (presence of reef-cage or not or the type of reef substrate), season (2 levels: spring vs autumn) and the influence of the time of day for a net set (2 levels: day vs night) as fixed factors, and site as a random factor (S1 File). Spring was defined as catches from April, May and June; while Autumn included catches from August, September, and October. Post-hoc effects were evaluated for those models that included significant effects of cage treatment and diurnal activity (day/night), using the emmeans package in R (aka Least-Squares Means; Lenth 2023 Second, the effects of the presence of the reef-cages and type of reef-cage on species composition of fish was evaluated with multivariate analysis (PERMANOVA) using the vegan package version 2.6–4 [44; S2 File]. This analysis only included traps with fish in the catch (reducing the dataset from 473 to 55 samples). The multivariate model included a full factorial design of the fixed factors reef presence (2 levels: reef-cage vs no-reef cage) or reef-type (6 levels: cockle shells vs cobbles vs pebbles vs wood vs concrete vs BESE structures), season (2 levels: spring vs autumn) and time of day (2 levels: day vs night). In the latter analysis of reef-cage type, we did not include the no-cage treatment, further reducing the dataset to 43 samples with fish. The multivariate data was further explored with a Similarity Percentages analysis (SIMPER) to pinpoint the species that contributed most to the variation in catch [44]. We also performed a redundancy analysis on transformed data to visually explore the effects of the reef-cages better [S2 File; 44].

## Results

### Fish catch—Traps

**Fig 3:**
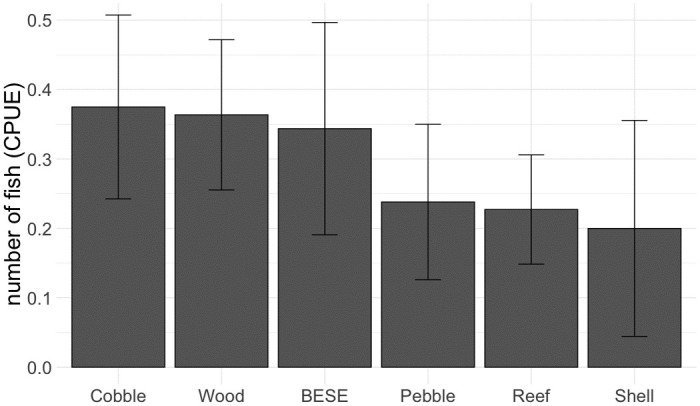
Mean counts of fish caught per unit effort (± SE) per reef type. Catch per unit effort (CPUE) is expressed as number of fish caught in each trap per 12 hours of fishing.

A total of 81 fish from 16 different species were caught in a total of 473 set traps across all fishing trips on reef-cages and no-reef controls (Table 1). There were no significant differences in catch of fish between the different reef types, neither for abundance (GLM: χ^2^ = 1.98, p = 0.852), species richness (GLM: χ^2^ = 3.38, p = 0.642) or community composition (PERMANOVA: F = 0.9, p = 0.650; S2 File; Fig 3), and therefore further comparisons focus on the effects of the reefs by aggregating all different reef structures to ‘reef-cage’ versus ‘no-reef control’.

**Table 1:** Species and numbers of individuals caught on reef-cages and in reference traps. A total of 227 traps were set on reef cages and 252 traps on reference lines.

Species Common Name	*Latin name*	Total catch on reef-cage	Total catch in no-reef reference
**European eel**	*Anguilla anguilla*	6	
**Five-bearded rockling**	*Ciliata mustela*	34	8
**European seabass**	*Dicentrarchus labrax*	1	
**Atlantic cod**	*Gadus morhua*	1	
**Goby spp**.	*Gobiidae spp*.	4	
**Common blenny**	*Lipophrys pholis*	1	
**Whiting**	*Merlangius merlangus*	1	
**Bullrout**	*Myoxocephalus scorpius*	2	
**European smelt**	*Osmerus eperlanus*	1	
**Rock gunnel**	*Pholis gunnellus*	4	1
	Flatfish spp.	9	2
**European flounder**	* Platichthys flesus*		
**European plaice**	* Pleuronectes platessa*		
**Common sole**	*Solea solea*		1
**Greater pipefish**	*Sygnathus acus*	1	
**Lesser pipefish**	*Syngnathus rostellatus*		2
**Eelpout**	*Zoarces viviparus*	1	1

**Fig 4:**
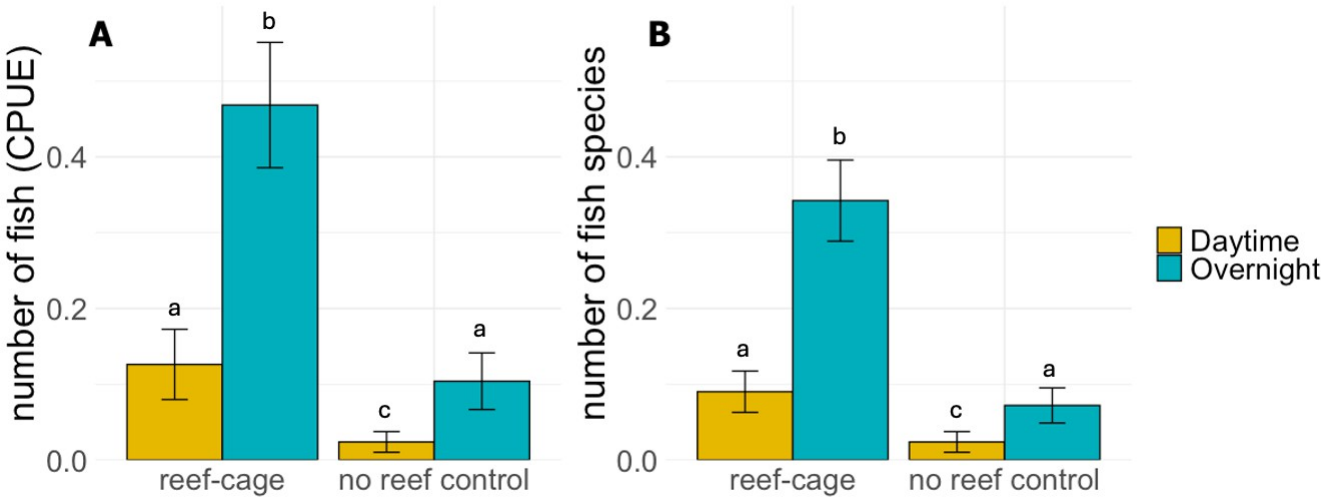
(A) Mean counts of fish ± SE and (B) mean number of fish species ± SE within traps on reef cages and on reference sets for daytime (yellow bars) and overnight (blue bars) catches. Catch per unit effort (CPUE) is expressed as number of fish caught in each trap per 12 hours of fishing. Different letters denote significant post-hoc contrasts (S1.5 Table in S1 File).

Although the number of fish caught per trap was relatively low, the mean number of fish caught on reef cages was still nearly five times higher than in the no-reef control traps (Generalized Linear Mixed Model: main effect of reef-cage, t = 4.5, p<0.001; Fig 4A). The mean catch was four times higher overnight compared to during the day (GLMM: main effect of day vs night fishing, t = 3.9, p<0.001; Fig 4A) and two times higher in the late summer/autumn compared to spring/early summer (GLMM: main effect of season, t = 2.8, p = 0.004; 0.24±0.08 fish per trap in late summer/autumn and 0.11±0.06 fish per traps in spring/early summer; mean±ci). There were no significant interaction effects between reef-cage/no-reef control, day-night catch or season included in the best statistical model (S1.1 Table in S1 File).

Apart from the difference in abundances, there were also more species of fish caught on the reef cages than on the sediment in the no-reef controls. In total, 14 species were caught on the reef cages and 6 species in the no-reef control (Table 1). The average number of fish species per trap was 4.5 times higher on the reefs (Generalized Linear Mixed Model: main effect of reef cage, t = 4.9, p<0.001; Fig 4B). The mean number of species caught was higher overnight than during the day (GLMM: main effect of day vs night fishing, t = 4.3, p<0.001). There were no seasonal differences in the number of species and there were no significant interaction effects included in the best statistical model (S1.2 Table in S1 File).

**Fig 5:**
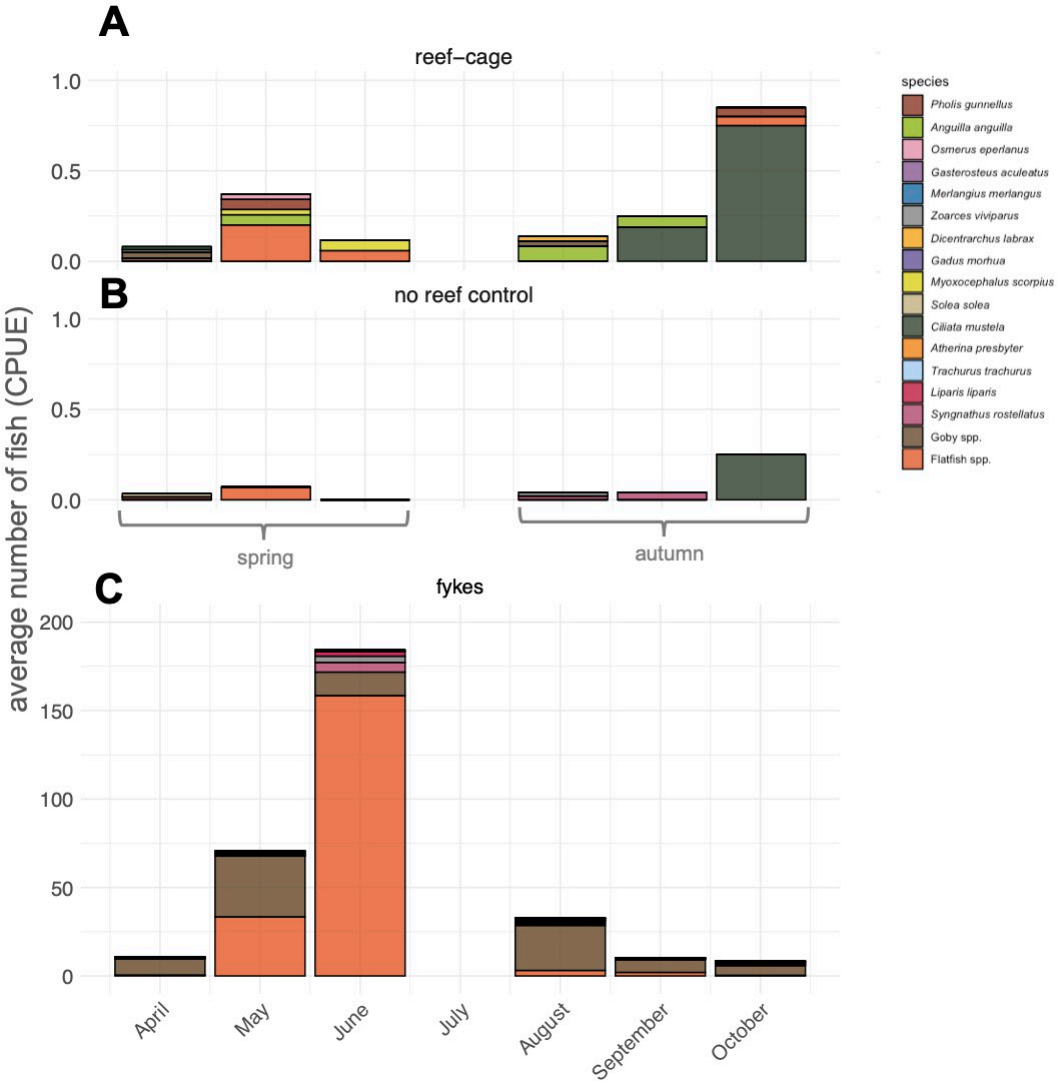
Fish species composition on (A) reef-cages and (B) no reef controls during spring (left), and autumn (right) fishing events between April 2021 to October 2021. (C) Fish species composition in fyke catches April 2021 to October 2021. Catch per unit effort (CPUE) is expressed as number of fish caught in each net per 12 hours of fishing.

In contrast, the species composition of fish was significantly affected by season, but not by reef treatments or by day/night catch catch (PERMANOVA: only the main effect of season was significant, F_1,47_ = 10.3, p<0.001; S2.1 Table in S2 File). Five-bearded rockling (*Ciliata mustela*), and juvenile flatfish (*Platichthys flesus* and *Pleuronectes platessa*) contributed most to the dissimilarity between spring and autumn (SIMPER, average contribution rockling: 35.2%, p = 0.032; flatfish: 18.2%, p<0.001; S2.2 Table in S2 File). In spring, juvenile flatfish were the primary catch and were three times more common in the reef traps compared to the no-reef control traps (0.07 ± 0.03 fish per trap on reef-cages and 0.025 ± 0.03 fish per trap on no-reef controls, mean ± SE; Mann-Whitney-Wilcoxon Test [data not suitable for GLMM], W = 7080.5, p-value = 0.051; Fig 5A & 5B). In autumn, five-bearded rockling (*Ciliata mustela*) were most abundant, and they were five times more common in the reef traps (0.31 ± 0.16 *C*. *mustela* per trap on reef-cages and 0.06 ± 0.05 *C*. *mustela* per trap in no-reef control sets, mean ± ci; Generalized Linear Mixed Model: main effect of reef-cage, t = 3.2, p<0.01, Fig 5A & 5B).

### Fish catch—Fykes

In the fyke nets, flatfish were the most common in the spring/early summer catches (Fig 5C). However, five-bearded rockling were almost absent from the fyke catches (n = 7 over all sets across seasons), and the late summer/autumn community was instead dominated by goby species (common goby (*Pomatoschistus microps*) and sand goby (*Pomatoschistus minutus*); Fig 5C). Several large catches of Atlantic herring (*Clupea harengus*) were removed from the dataset for the interpretation of this data as these schools can occur haphazardly.

### Invertebrate catch—Traps

**Fig 6:**
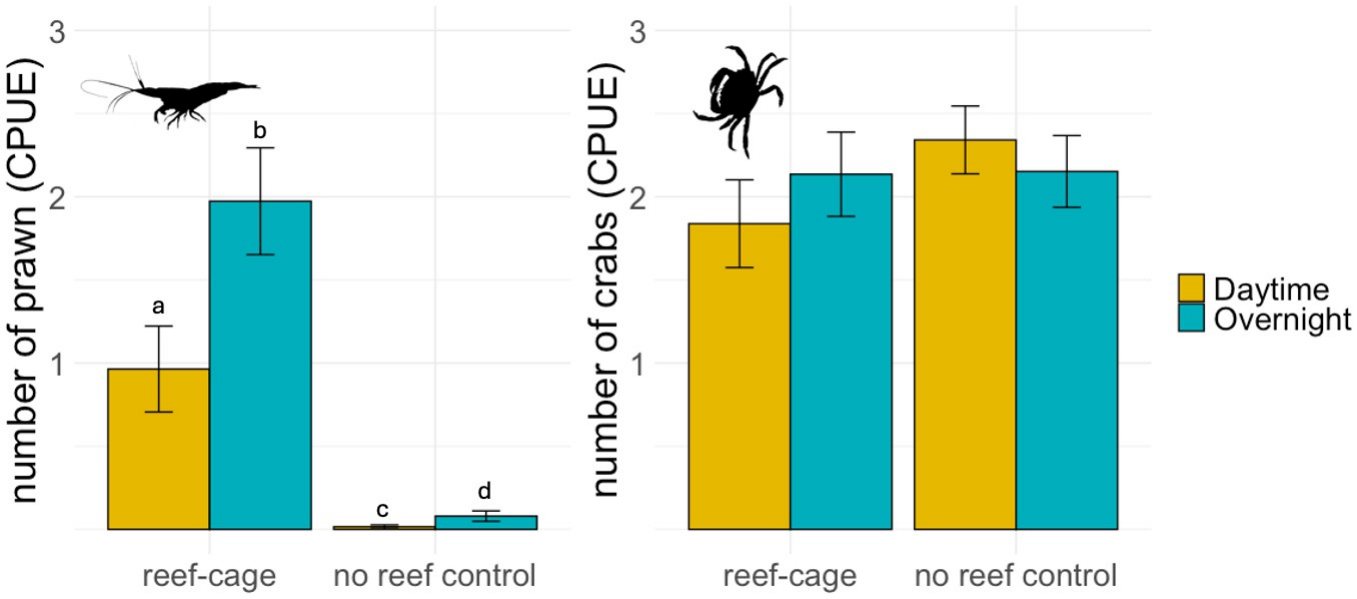
Mean number of A) prawn (± SE), and B) crab caught within traps on reef cages and on reference sets for daytime (yellow bars) and overnight (blue bars) catches. Catch per unit effort (CPUE) is expressed as number caught in each trap per 12 hours of fishing. Different letters denote significant post-hoc contrasts (S1.5 Table in S1 File).

Catch of prawn (*Palaemon* spp.) were far greater in the traps at the reef cages than in the controls; in total 326 individuals were caught on the cages and only 12 in the no-reef control traps (GLMM: main effect of reef, t = 7.9, p<0.001; Fig 6A). The catch was also higher overnight (GLMM: main effect of day vs night fishing, t = 4.3, p<0.001; Fig 6A) and in autumn compared to spring (GLMM: main effect of season, t = 2.8, p = 0.004; 0.89±0.34 prawn per trap in late summer/autumn and 0.53±0.21 prawn per traps in spring/early summer; mean±ci), but there were no significant interaction effects included in the best fitting model (S1.3 Table in S1 File). European Green crab (*Carcinus maenas*) was more common in autumn than in spring (GLMM: main effect of season, t = 4.8, p<0.001; 3.0±0.37crab per trap in late summer/autumn and 1.2±0.21 crab per traps in spring/early summer; mean±ci). But although the reef treatment was included as an interaction with season in the best selected model (S1.4 Table in S1 File), there was no influence of the reef-cages on catches of crabs (GLMM: main effect of reef treatment, t = 1.5, p = 0.139; interaction effect season x reef treatment, t = 0.9, p = 0.382; Fig 6B). There were no significant differences in catch of prawn or crab between the different reef types (GLM, main effect of cage type on: prawn, χ^2^ = 3.26, p = 0.660 crabs, χ^2^ = 3.82, p = 0.575; S1.2 Fig in S1 File).

## Discussion

Artificial reefs are recognized as an effective method to enhance fish habitat but results are highly dependent on the local environment and reef design [19]. Here we demonstrate that the introduction of hard substrates in the subtidal Wadden Sea attracts a distinct mobile community compared to adjacent soft-sediment habitats. Hard substrate habitats (such as boulder fields, sunken wood aggregations and European oyster reefs) were historically much more common in the Wadden Sea but have declined dramatically or disappeared completely due to human engineering, bottom trawling and dredging [13, 27, 35, 37, 38]. At the same time, fish communities are fundamentally changed and currently the function of the Wadden Sea as a nursery habitat for many fish species is in decline [12, 13, 45]. The species found on the reef cages were distinct from the control catches and from the larger scale fyke catches in the same gullies. These results show that structure-dependent communities of mobile species respond quickly and positively to the re-introduction of reef structures into this degraded environment, and suggesting that restoration of hard substrates is a relevant strategy to support these communities. These results may therefore motivate nature management organizations to consider larger-scale reef restoration and artificial reef design in degraded temperate marine environments [46].

Introduction of artificial reefs increases local abundance and diversity of mobile species through creation of habitat complexity, and may build communities that are distinct from naturally occurring reefs in the same area [22, 47, 48]. The distinct assemblage of fish species caught on the reef-cages included species known to prefer more complex habitat structures such as the five-bearded rockling (*C*. *mustela)*, European eel (*Anguilla anguilla*) and shorthorn sculpin (*Myoxocephalus scorpius*). Other fish species exclusively found in reef-cage traps included commercial fish such as Atlantic cod (*Gadus morhua*), European seabass (*Dicentrarchus labrax*), and whiting (*Merlangius merlangus*). Understanding how fish species use different habitats within the Wadden Sea has been identified as a critical knowledge gap for decision frameworks aimed at managing the fish community [49]. We did not find any difference in fish abundances or diversity between the substrate types, suggesting that for these mobile species, the presence of a hard substrate or reef structure is a more important factor over the specific structural component itself at small scales. While we may have expected interactions between reef-cage presence and day/night activity due to known diel patterns in fish behavior, the strong influence of structure itself may have overshadowed any variation associated with time of day.

Introductions of hard substrates for restoration and habitat improvement for fish should have clear objectives for the types of habitats that are being restored and their functions for the fish community. Where reef type and habitat suitability are not accounted for, substrates or artificial reef introductions may change, rather than restore, the community. Since these long-term effects are highly context dependent, we need to monitor species’ and community responses over time and in different systems to determine the broader effects of substrate introductions [19, 20].

Rapid aggregation of fish is a common but not always certain outcome of artificial reef introduction across tropical and temperate projects [7, 22, 30, 50]. This has sparked ongoing discussions on how to quantify attraction versus production of fish biomass at these sites [16, 51], and what implications attraction of fish has for adjacent natural habitats. Successional changes in mobile communities at introduced reef structures may also occur as communities at other trophic levels develop [52]. We sampled across all seasons over a year and half after the deployment of the substrates, which revealed the rapid initial arrival of mobile species. The temporal changes in the composition of fish species and crab abundances followed documented seasonal changes in the area, with flatfish dominating in spring and five-bearded rockling in autumn. The seasonality of fish and crabs closely followed local changes in water temperature which commonly triggers both species activity patterns and seasonal migrations of specific guilds to and from the Wadden Sea [53–56; S1.3 Fig in S1 File]. However, the monitoring period for this study was too short to evaluate further succession of the reef-associated mobile communities. To acquire information on these longer-term local fish population trends, it is necessary to include longer term monitoring.

Globally, concrete is the predominant material used in the construction of artificial reefs [19, 46]. Such concrete structures have been shown to be highly successful in restoring habitat complexity, with levels of complexity comparable to local natural reefs [19]. However, these designs may not be successful in all locations if consider the local ecosystem features and ecological context [57]. The results of our study show that, from the perspective of mobile species, other materials could also be used, since species respond similarly to different substrate types. In larger restoration efforts, we could therefore use more natural or biodegradable materials that facilitate, and be replaced by, reef-building species rather than long-lasting artificially produced substrates [58, 59].

The fish community along the coastal zone of the Netherlands is changing and reports indicate that important species of fish may be escaping warming waters [45, 60, 61]. The results from this study show seasonal changes in reef habitat use by different species. We observed shifts in dominant species caught at the reef-cages, from flatfish species in spring to *C*. *mustela* in autumn. Catches in the fykes were dominated by flatfish and goby species and also showed a seasonal rise in flatfish numbers in May and June. These shifts are consistent with the knowledge that juvenile flatfish use the Wadden Sea as a nursery area in the spring and summer and move offshore in the autumn [62, 63]. Notably, *C*. *mustela* were found in very low numbers in the fyke catches throughout seasons. The preference of *C*. *mustela* for more complex substrates has been shown in laboratory experiments that this species will tolerate higher water temperatures to stay within sheltered habitat [64]. This supports studies showing that fish that are associated with bottom hard substrates are less susceptible to climate change-driven effects [65]. Our results demonstrate that many mobile organisms prefer sheltered habitats, which suggests that restoring reefs in the Wadden Sea may be an effective strategy to mitigate negative effects of climate change for fish associated with hard substrates.

Fish communities at both introduced and natural reefs are highly influenced by local environmental conditions, and their relation to other natural habitats within the broader ecosystem [22, 66, 67]. On a seascape level, loss of complex habitats like shellfish reefs, macroalgal beds or seagrass can result in system-wide changes to the mobile communities that rely on different habitats [68] and may also impede or prohibit recovery of declining fish populations [69]. Here, the site of deployment did not have a significant influence on the numbers of fish or prawn, showing that the introduction of hard substrates had a similar effect across different tidal channels. In the Wadden Sea, fish use habitats as part of their migratory network to and from spawning grounds, nursery areas, and stop-over areas [45]. However, for many species that have been recorded in this region, information on habitat use and seasonal movements are largely unknown [15]. Further understanding of reef communities in the Dutch Wadden Sea will require examining how these species use and move between habitats, the relative importance of habitat types for different species, and the function of different habitats at different life-stages.

The loss of naturally occurring hard substrates such as rock and wood mean that restoration of such benthic habitats in the Dutch Wadden Sea will likely require active management in order to support the communities which rely on these substrates and more complex habitat structure. Artificial reef introductions pose a promising method to facilitate the functions of biogenic reefs for mobile species. The outcomes of this study show that even at small scales, the introduction of habitat complexity via hard substrates can be successful for supporting mobile communities. This highlights the potential for scaling-up habitat restoration through increasing substrate complexity and heterogeneity in coastal systems like the Wadden Sea.

## Supporting information

S1 File Tables with terms and results from generalized linear models with AIC values, fish abundances during reef cage lifting monitoring, and average salinity and temperature values during sampling events. (DOCX)

S2 File Detailed methods of multivariate analyses testing the effect of different reef-cage types on the composition of fish species across seasons. (DOCX)

S3 File Species counts per catch for all fyke catches across sites and seasons. (CSV)

S4 File Species counts for all trap (kubben) catches across sites and seasons. (CSV)
