# Supplementary material for: Introduced substrates trigger colonization by reef-associated fish in a degraded coastal system
Source: PLoS One. 2025 Jan 30;20(1):e0317431. doi: 10.1371/journal.pone.0317431 (PMC11781683; doi:10.1371/journal.pone.0317431)
Supplement: S1 File — (DOCX) [file pone.0317431.s001.docx]

# SUPPLEMENTARY MATERIAL 1

Table S1.1. Generalized linear model terms for the fish abundance models and their AIC values, Site refers to the different reef-cage blocks, Treatment Type is on reef-cages or on reference sets.

|  | Model terms | df | AIC |
| --- | --- | --- | --- |
| 1 | Total Fish ~1\|site | 2 | 501.663 |
| 2 | Total fish ~ Treatment Type + 1\|site | 4 | 478.9849 |
| 3 | Total fish ~ Day/Night + 1\|site | 4 | 482.9852 |
| 4 | Total fish ~ Season + 1\|site | 4 | 498.0403 |
| 5 | Total fish ~ Treatment Type +Day/Night + 1\|site | 7 | 459.0042 |
| 6 | Total fish ~Treatment Type + Season + 1\|site | 7 | 472.2005 |
| **7** | **Total fish** ~ **Treatment Type + Day/Night + Season + 1\|site** | **11** | **452.6783** |
| 8 | Total fish ~ Treatment Type *Day/Night *Season + 1\|site | 37 | 493.1042 |

Table S1.2. Generalized linear model terms for the fish species richness models and their AIC values, Site refers to the different reef-cage blocks, Treatment Type is on reef-cages or on reference sets.

|  | Model terms | df | AIC |
| --- | --- | --- | --- |
| 1 | Richness ~1\|site | 2 | 377.7013 |
| 2 | Richness ~ Treatment Type + 1\|site | 4 | 369.01310 |
| 3 | Richness ~ Day/Night + 1\|site | 4 | 370.5800 |
| 4 | Richness ~ Season + 1\|site | 4 | 380.3566 |
| **5** | **Richness ~ Treatment Type + Day/Night + 1\|site** | **7** | **356.1616** |
| 6 | Richness ~ Treatment Type + Season + 1\|site | 7 | 368.9961 |
| 7 | Richness ~ Treatment Type + Day/Night + Season + 1\|site | 11 | 358.8154 |
| 8 | Richness ~ Treatment Type *Day/Night *Season + 1\|site | 37 | 406.0216 |
| 9 | Richness ~ Treatment Type *Day/Night + 1\|site | 11 | 363.9820 |

Table S1.3. Generalized linear model terms for the prawn abundance models and their AIC values, Site refers to the different reef-cage blocks, Treatment Type is on reef-cages or on reference sets.

|  | Model terms | df | AIC |
| --- | --- | --- | --- |
| 1 | Total Prawn ~1\|site | 2 | 1410.4890 |
| 2 | Total Prawn ~ Treatment Type + 1\|site | 4 | 1040.6597 |
| 3 | Total Prawn ~ Day/Night + 1\|site | 4 | 1351.1034 |
| 4 | Total Prawn ~ Season + 1\|site | 4 | 1324.8352 |
| 5 | Total Prawn ~ Treatment Type +Day/Night + 1\|site | 7 | 980.6972 |
| 6 | Total Prawn ~ Treatment Type + Season + 1\|site | 7 | 954.3007 |
| **7** | **Total Prawn** ~ **Treatment Type + Day/Night + Season + 1\|site** | **11** | **894.8584** |
| 8 | Total Prawn ~ Treatment Type *Day/Night *Season + 1\|site | 37 | 928.8678 |

Table S1.4. Generalized linear model terms for the crab abundance models and their AIC values, Site refers to the different reef-cage blocks, Treatment Type is on reef-cages or on reference sets.

|  | Model terms | df | AIC |
| --- | --- | --- | --- |
| 1 | Total Crabs ~ 1\|site | 2 | 2134.786 |
| 2 | Total Crabs ~ Treatment Type + 1\|site | 4 | 2097.165 |
| 3 | Total Crabs ~ Day/Night + 1\|site | 4 | 2134.666 |
| 4 | Total Crabs ~ Season + 1\|site | 4 | 1980.391 |
| 5 | Total Crabs ~ Treatment Type +Day/Night + 1\|site | 7 | 2097.865 |
| 6 | Total Crabs ~ Treatment Type + Season + 1\|site | 7 | 1949.837 |
| 7 | Total Crabs ~ Treatment Type + Day/Night + Season + 1\|site | 11 | 1953.797 |
| 8 | Total Crabs ~ Treatment Type *Day/Night *Season + 1\|site | 37 | 1951.733 |
| **9** | **Total Crabs ~ Treatment Type *Day/Night + 1\|site** | **11** | **1945.408** |

| Table S1.5: post hoc contrasts between significant GLM effects; calculated using Least-Squares Means (Lenth 2023). | | | | |
| --- | --- | --- | --- | --- |
|  |  |  | t | p |
| Abundance of fish: |  |  |  |  |
| artificial reef daytime | vs | no reef daytime | 4.5 | <0.001 |
| artificial reef daytime | vs | artificial reef overnight | 3.9 | <0.001 |
| artificial reef daytime | vs | no reef overnight | 0.5 | 0.966 |
| no reef daytime | vs | artificial reef overnight | 6.0 | <0.001 |
| no reef daytime | vs | no reef overnight | 3.9 | <0.001 |
| artificial reef overnight | vs | no reef overnight | 4.5 | <0.001 |
|  |  |  |  |  |
| Species richness of fish: |  |  |  |  |
| artificial reef daytime | vs | no reef daytime | 4.6 | <0.001 |
| artificial reef daytime | vs | artificial reef overnight | 4.3 | <0.001 |
| artificial reef daytime | vs | no reef overnight | 0.5 | 0.955 |
| no reef daytime | vs | artificial reef overnight | 6.6 | <0.001 |
| no reef daytime | vs | no reef overnight | 4.3 | <0.001 |
| artificial reef overnight | vs | no reef overnight | 4.9 | <0.001 |
|  |  |  |  |  |
| Abundance of shrimp: |  |  |  |  |
| artificial reef daytime | vs | no reef daytime | 7.9 | <0.001 |
| artificial reef daytime | vs | artificial reef overnight | 4.3 | <0.001 |
| artificial reef daytime | vs | no reef overnight | 5.7 | <0.001 |
| no reef daytime | vs | artificial reef overnight | 8.9 | <0.001 |
| no reef daytime | vs | no reef overnight | 4.3 | <0.001 |
| artificial reef overnight | vs | no reef overnight | 7.9 | <0.001 |


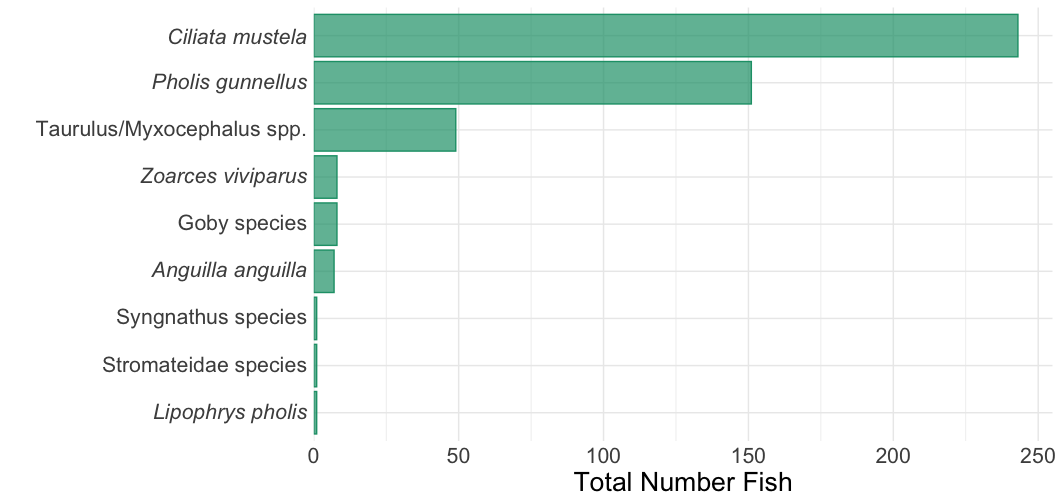


Figure S1.1. Total abundance of fish found on or within reef cages during lifting events for sessile species surveys. A total of 469 fish belonging to at least 10 species (some fish were identified to family) were found on, or fell out of, reef cages during lifting of the reef cages for benthic sampling. The most common species found during these events was *Ciliata mustela* (243 individuals) and *Pholis gunnelus* (151 individuals.


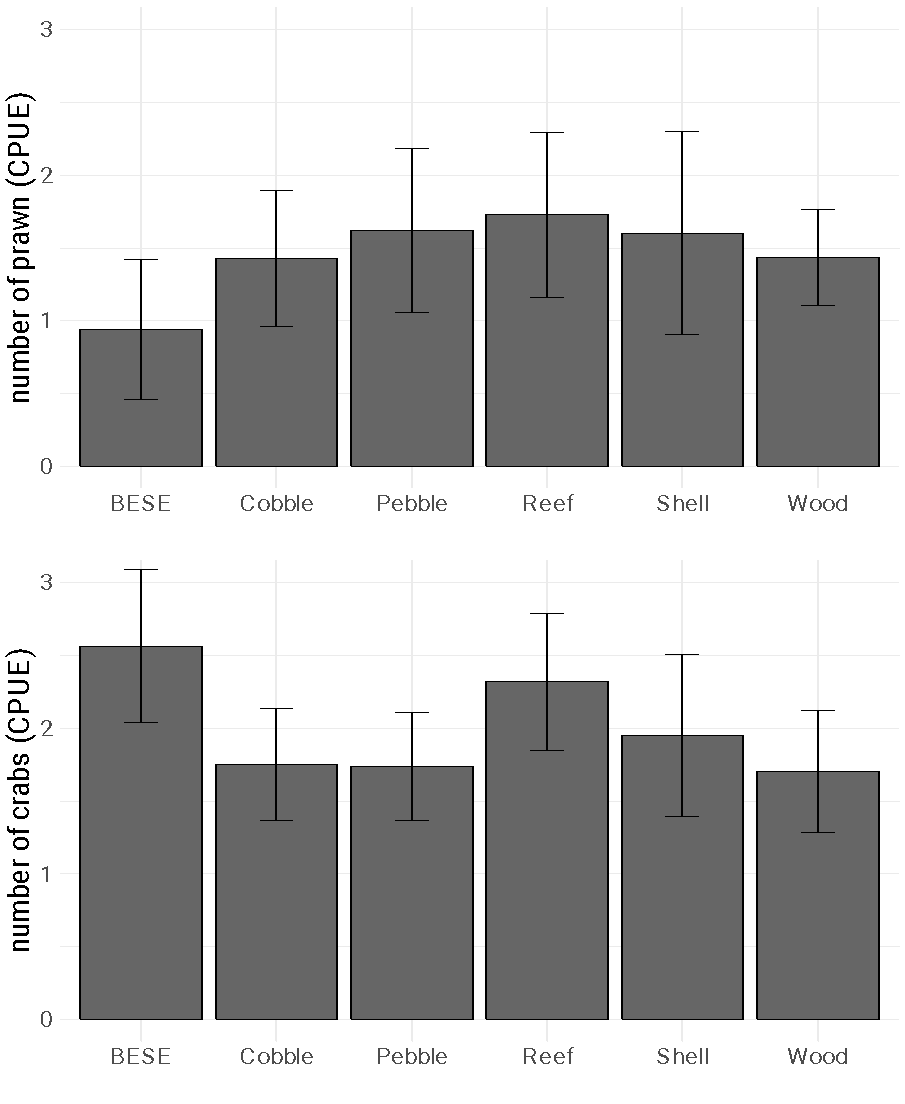


**B**

**A**

Figure S1.2. Mean (A) prawn and (B) crab catches (± SE) at the reef-cages by the reef type.


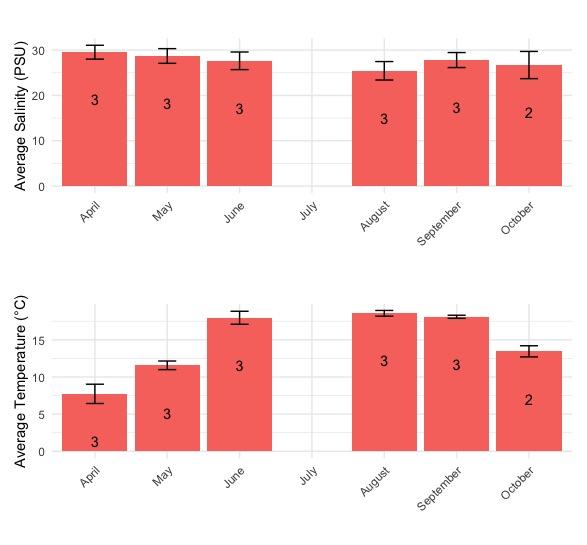


Figure S1.3. Average salinity (PSU) and temperature (oC) during the fishing events months (April 2021 - October 2021). Salinity and temperature measurements from Rijkswaterstaat Waterinfo stations. Numbers on the bars indicate the number of measurements per month.
