# Supplementary material for: Introduced substrates trigger colonization by reef-associated fish in a degraded coastal system
Source: PLoS One. 2025 Jan 30;20(1):e0317431. doi: 10.1371/journal.pone.0317431 (PMC11781683; doi:10.1371/journal.pone.0317431)
Supplement: S2 File — (DOCX) [file pone.0317431.s002.docx]

Supplement 2: Multivariate analyses testing the effect of the different reef-cage types on the composition of fish species across seasons.

*Methods*

The effects on the composition of fish by adding reef-cages was evaluated with Permutational Multivariate Analysis of Variance (PERMANOVA) using the vegan package version 2.6-4 (Oksanen et al. 2022). Since the data had a non-parametric distribution with heteroscedastic variances, distances between samples were calculated using Bray-Curtis dissimilarities (non-parametric method). There was a high number of empty traps throughout, which is challenging to handle with multivariate analyses (Buckley et al. 2021). We therefore started by removing all the traps without any catch of fish from the analysis, reducing the dataset from 473 to 55 samples (McCune and Grace 2002). We performed two separate analysis: one that focused on testing the effect of placing a reef-cage on the bottom or not (2 levels: reef-cage vs no-cage); and one focusing on testing the effect of the different types of reef (6 levels: cockle shells vs cobbles vs pebbles vs wood vs concrete vs BESE structures). In the latter analysis we did not include the no-cage treatment, further reducing the dataset to 43 samples with fish. The model testing the effect of placing a reef-cage (or not) included a full factorial combination of the fixed factors reef-cage treatment (presence of cage or not), season (2 levels: spring vs autumn) and time of day (2 levels: day vs night). The model testing the effect of reef-type only included 2-way interactions, due to the limited replication of individual reef-types. The PERMANOVA’s were further explored with a Similarity Percentages analysis (SIMPER) to pinpoint the species that contributed most to the differences in catch between groups of significant factors (Oksanen et al. 2022).

We also performed a redundancy analysis (RDA) on transformed data (using the vegan package version 2.6-4, Oksanen et al. 2022) to visually explore the effects of the reef-cages and check if a parametric method would be stronger in picking up cage effects. Note that the data is better analysed with the non-parametric PERMANOVA and that this analysis was mainly to check the robustness of the previous analysis and to produce a plot with constrained ordination axes. Also here we performed two separate analysis: one that focused on testing the effect of placing a reef-cage on the bottom or not (2 levels: reef-cage vs no-cage), removing all samples without any catch of fish from the analysis; and one focusing on testing the effect of the different types of reef (6 levels: cockle shells vs cobbles vs pebbles vs wood vs concrete vs BESE structures) were we also removed the samples belonging to the no-reef cage treatment. We reduced the models from the start to only include the already persuasive effects of season (spring vs autumn) and the experimental reef-cage treatments (reef-cage vs no-reef cage; or type of cage); to constrain the analysis along two ordination axes only. We tested the significance of the different constraining factors using the function anova.cca and then tested the fit of each species to the two ordination axes using the envfit function (using the vegan package version 2.6-4, Oksanen et al. 2022).

*Results and conclusion*

The PERMANOVA testing effects of the presence of a reef-cage on the bottom or not, showed that the species composition of fish was significantly affected by season, but not by the presence of a reef-cage or by the time of the day of the catch (Table S2:1). The SIMPER analysis showed that juvenile flatfish (*Platichthys flesus* and *Pleuronectes platessa*) dominated in spring and five-bearded rockling (*Ciliata mustela*) dominated in the autumn; together contribution to more than 50 % to the dissimilarity in species composition between spring and autumn (Table S2:2). Rock gunnel and bullrout also contributed significantly to the differences between spring and autumn (for bullrout it was a marginal trend), but to a much lesser extent.

The PERMANOVA testing effects of type of reef-cage (excluding the no-reef cages), similarly showed that the species composition of fish was significantly affected by season, but not by the type of reef-cage or by the time of the day of the catch (Table S2:3). The SIMPER analysis on the reduced dataset also showed that juvenile flatfish (*Platichthys flesus* and *Pleuronectes platessa*) dominated in spring and five-bearded rockling (*Ciliata mustela*) dominated in the autumn; together contribution to more than 50 % to the dissimilarity in species composition between spring and autumn (S2:4). However, in the reduced model, no other species contributed significantly to the seasonal differences.

The RDA constrained by the factors season and the presence of a reef-cage or not, explained 19.9 % of the total variation in fish community composition. The constraining factor season alone significantly explained 18.5 % of the variation in species data (F=12.1, p<0.001) and related strongly to ordination axis 1 (Biplot score for spring along RDA 1 was positive 0.99; Fig. S2:1). The constraining factor reef-cage related strongly to ordination axis 2 (RDA 2; Fig. S2:2), but did not significantly explain any of the variation in species data (F=0.86, p=493). The abundance of eelpout, European eel, five-bearded rockling, juvenile flatfish and lesser pipefish were significantly described by the ordination axes (Table S2:5, Fig. S2:3). Five-bearded rockling and juvenile flatfish showed a strong association to autumn and spring, respectively: rockling had the lowest score along RDA 1 and juvenile flatfish had the highest score along RDA 1 (Table S2:5, Fig. S2:3).

The RDA constrained by the factors season and the type of reef, explained 27.7 % of the total variation in fish community composition (full model: F=2.3, p=0.002). The constraining factor season alone significantly explained 20.3 % of the variation in species data (F=10.1, p<0.001) and related strongly to ordination axis 1 (Biplot score for spring along RDA 1 was negative 0.99; Fig. S2:4). The constraining factor reef type related strongly to ordination axis 2 (RDA 2; Fig. S2:5), but did not significantly explain any of the variation in species data (F=0.74, p=814). Five-bearded rockling and juvenile flatfish showed a strong association to autumn and spring, respectively: rockling had the highest score along RDA 1 and juvenile flatfish had the lowest score along RDA 1 (Table S2:6, Fig. S2:6).

Thus, the RDA analysis strongly supports the PERMANOVA and GLM results, confirming that season drives the patterns in community composition and that the effects of the cages are mainly driven by changes in number and diversity of fish.

| Table S2.1: PERMANOVA effect on the composition of the fish community depending on presence of cage-reefs or not, time of day and season. | | | | |
| --- | --- | --- | --- | --- |
|  | df | R^2^ | F | p |
| Reef-cage (or not) | 1 | 1.8 | 1.2 | 0.230 |
| Time of day (day or night catch) | 1 | 2.1 | 1.4 | 0.169 |
| Season (spring or autumn) | 1 | 15.5 | 10.0 | < 0.001 |
| Reef-cage x Time of day | 1 | 1.3 | 0.9 | 0.512 |
| Reef-cage x Season | 1 | 1.3 | 0.9 | 0.512 |
| Time of day x Season | 1 | 2.6 | 1.7 | 0.102 |
| Reef-cage x Time of day x Season | 1 | 2.2 | 1.4 | 0.179 |
| error | 47 |  |  |  |

| Table S2:2. SIMPER results showing the average contribution of each single species to the difference between spring and autumn (as demonstrated by a PERMANOVA); including all samples with fish. | | | |
| --- | --- | --- | --- |
| Common name | Latin name | Contribution (%) | p |
| Five-bearded rockling | *Ciliata mustela* | 35.2 | 0.026 |
| Flatfish spp. | *Platichthys flesus / Pleuronectes platessa* | 18.2 | 0.002 |
| Rock gunnel | *Pholis gunnellus* | 8.6 | 0.026 |
| European eel | *Anguilla anguilla* | 6.9 | 0.605 |
| Goby spp. | *Gobiidae spp.* | 6.5 | 0.103 |
| Bullrout | *Myoxocephalus scorpius* | 4.7 | 0.074 |
| Eelpout | *Zoarces viviparus* | 3.4 | 0.105 |
| Lesser pipefish | *Syngnathus rostellatus* | 2.4 | 0.613 |
| European smelt | *Osmerus eperlanus* | 2.4 | 0.259 |
| Greater pipefish | *Sygnathus acus* | 2.4 | 0.239 |
| Common sole | *Solea solea* | 2.4 | 0.263 |
| Atlantic cod | *Gadus morhua* | 1.2 | 0.439 |
| Common blenny | *Lipophrys pholis* | 1.2 | 0.442 |
| Whiting | *Merlangius merlangus* | 1.2 | 0.454 |
| European seabass | *Dicentrarchus labrax* | 0.8 | 0.426 |

| Table S2.3: PERMANOVA effect on the composition of the fish community depending on type of cage-reef (6 levels: cockle shells vs cobbles vs pebbles vs wood vs concrete vs BESE structures), time of day and season. | | | | |
| --- | --- | --- | --- | --- |
|  | df | R^2^ | F | p |
| Reef-type | 5 | 8.8 | 0.8 | 0.720 |
| Time of day (day or night catch) | 1 | 3.5 | 1.6 | 0.120 |
| Season | 1 | 17.0 | 8.0 | < 0.001 |
| Reef-type x Time of day | 3 | 6.8 | 1.1 | 0.397 |
| Reef-type x Season | 4 | 5.3 | 0.6 | 0.924 |
| Time of day x Season | 1 | 1.2 | 0.6 | 0.805 |
| error | 27 |  |  |  |

| Table S2:4. SIMPER results showing the average contribution of each single species to the difference between spring and autumn (as demonstrated by a PERMANOVA); only including the reef-cage samples (excluding the no-reef cage controls). | | | |
| --- | --- | --- | --- |
| Common name | Latin name | Contribution (%) | p |
| Five-bearded rockling | *Ciliata mustela* | 36.1 | 0.024 |
| Flatfish spp. | *Platichthys flesus / Pleuronectes platessa* | 16.9 | 0.003 |
| European eel | *Anguilla anguilla* | 8.6 | 0.590 |
| Goby spp. | *Gobiidae spp.* | 7.9 | 0.108 |
| Rock gunnel | *Pholis gunnellus* | 7.5 | 0.103 |
| Bullrout | *Myoxocephalus scorpius* | 5.6 | 0.104 |
| Eelpout | *Zoarces viviparus* | 2.8 | 0.309 |
| European smelt | *Osmerus eperlanus* | 2.8 | 0.299 |
| Greater pipefish | *Sygnathus acus* | 2.8 | 0.288 |
| Atlantic cod | *Gadus morhua* | 1.6 | 0.405 |
| Common blenny | *Lipophrys pholis* | 1.6 | 0.401 |
| European seabass | *Dicentrarchus labrax* | 1.6 | 0.384 |
| Whiting | *Merlangius merlangus* | 1.1 | 0.417 |

| Table S2.5. Fit of taxonomic groups to RDA ordination axes 1 and 2; based on constraining factors season and presence of reef-cage or not. | | | | |
| --- | --- | --- | --- | --- |
|  | RDA1 | RDA2 | R^2^ | p |
| Atlantic cod | 0.98 | -0.18 | 0.5 | 0.981 |
| bullrout | 0.99 | 0.04 | 2.4 | 0.587 |
| common blenny | 0.98 | -0.18 | 0.5 | 0.987 |
| common sole | 0.57 | -0.82 | 8.0 | 0.093 |
| **eelpout** | **0.61** | **-0.78** | **10.7** | **0.039** |
| **european eel** | **0.51** | **0.85** | **50.2** | **0.001** |
| european seabass | 0.98 | -0.18 | 0.5 | 0.988 |
| european smelt | 0.97 | -0.22 | 1.0 | 0.926 |
| **five-bearded rockling** | **-0.99** | **0.06** | **76.0** | **0.001** |
| **flatfish spp.** | **0.94** | **0.31** | **29.3** | **0.002** |
| **Goby spp.** | **0.73** | **0.68** | **9.5** | **0.059** |
| greater pipefish | 0.97 | -0.22 | 1.0 | 0.899 |
| **lesser pipefish** | **0.36** | **-0.93** | **38.6** | **0.001** |
| rock gunnel | 0.8 | -0.59 | 7.0 | 0.125 |
| whiting | -0.96 | 0.24 | 1.7 | 0.586 |

| Table S2.6. Fit of taxonomic groups to RDA ordination axes 1 and 2; based on constraining factors season and reef type. | | | | |
| --- | --- | --- | --- | --- |
|  | RDA1 | RDA2 | R2 | p |
| Atlantic cod | -0.57 | 0.81 | 4.0 | 0.478 |
| **Bullrout** | **-0.49** | **0.87** | **39.8** | **0.003** |
| Common blenny | -0.99 | -0.1 | 0.7 | 0.962 |
| Eelpout | -0.62 | 0.78 | 5.7 | 0.239 |
| **European eel** | **-0.64** | **-0.76** | **32.6** | **0.002** |
| European seabass | -0.99 | 0.14 | 0.5 | 0.979 |
| European smelt | -0.57 | 0.81 | 7.9 | 0.122 |
| **Five-bearded rockling** | **0.99** | **-0.03** | **76.6** | **0.001** |
| **Flatfish spp.** | **-0.98** | **-0.15** | **23.8** | **0.008** |
| **Goby spp.** | **-0.41** | **-0.91** | **24.6** | **0.003** |
| Greater pipefish | -0.57 | 0.81 | 7.9 | 0.105 |
| Rock gunnel | -0.35 | -0.93 | 5.9 | 0.308 |
| Whiting | 0.95 | -0.3 | 2.4 | 0.723 |


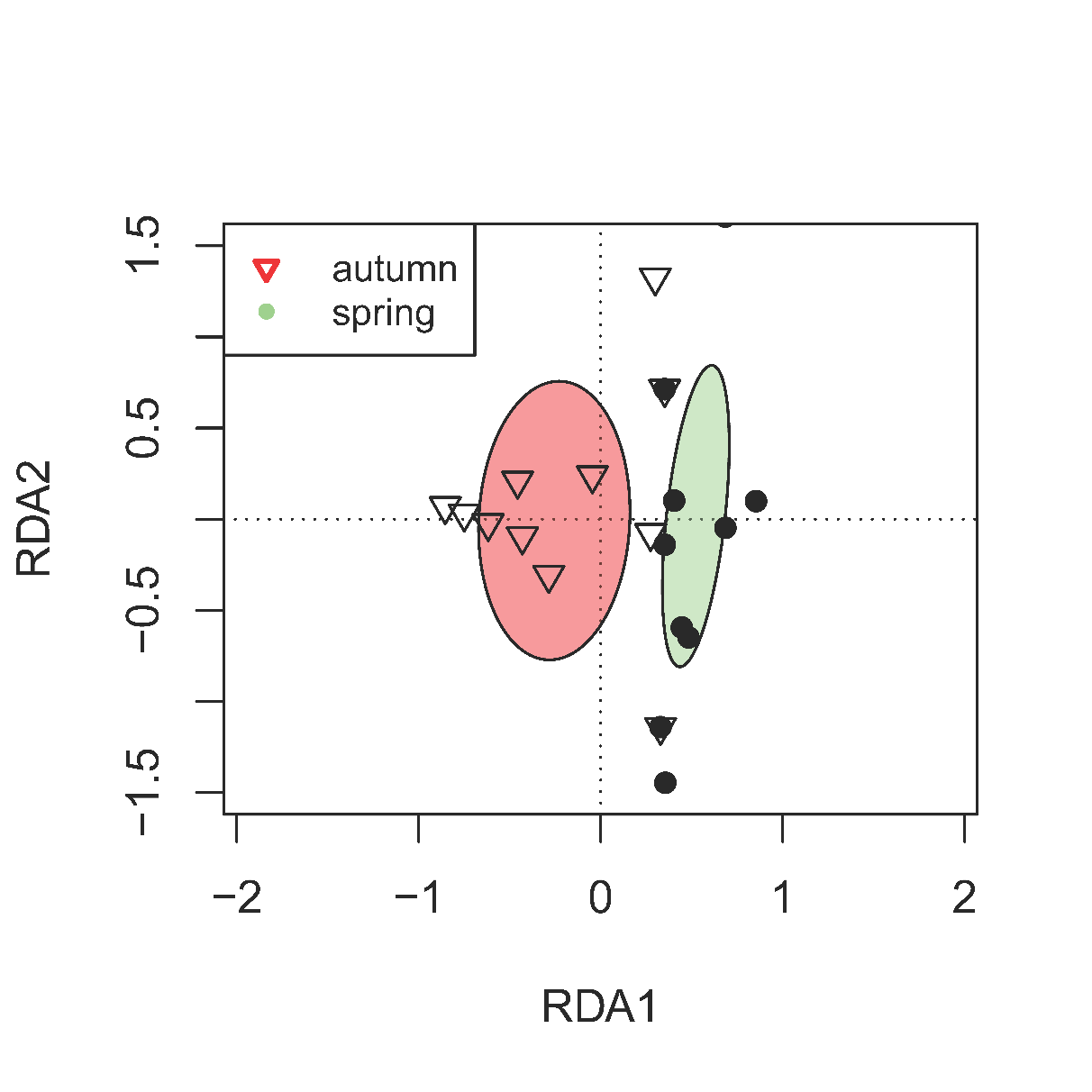


Figure S2:1. RDA plot showing sample scores and the separation of season along RDA axis 1. Triangles show the sample scores fro autumn and black dots show the sam[ple scores for spring. The red ellipse show the standard deviation around the centroid (mean) for autumn, the green ellipse show the standard deviation around the centroid (mean) for spring.


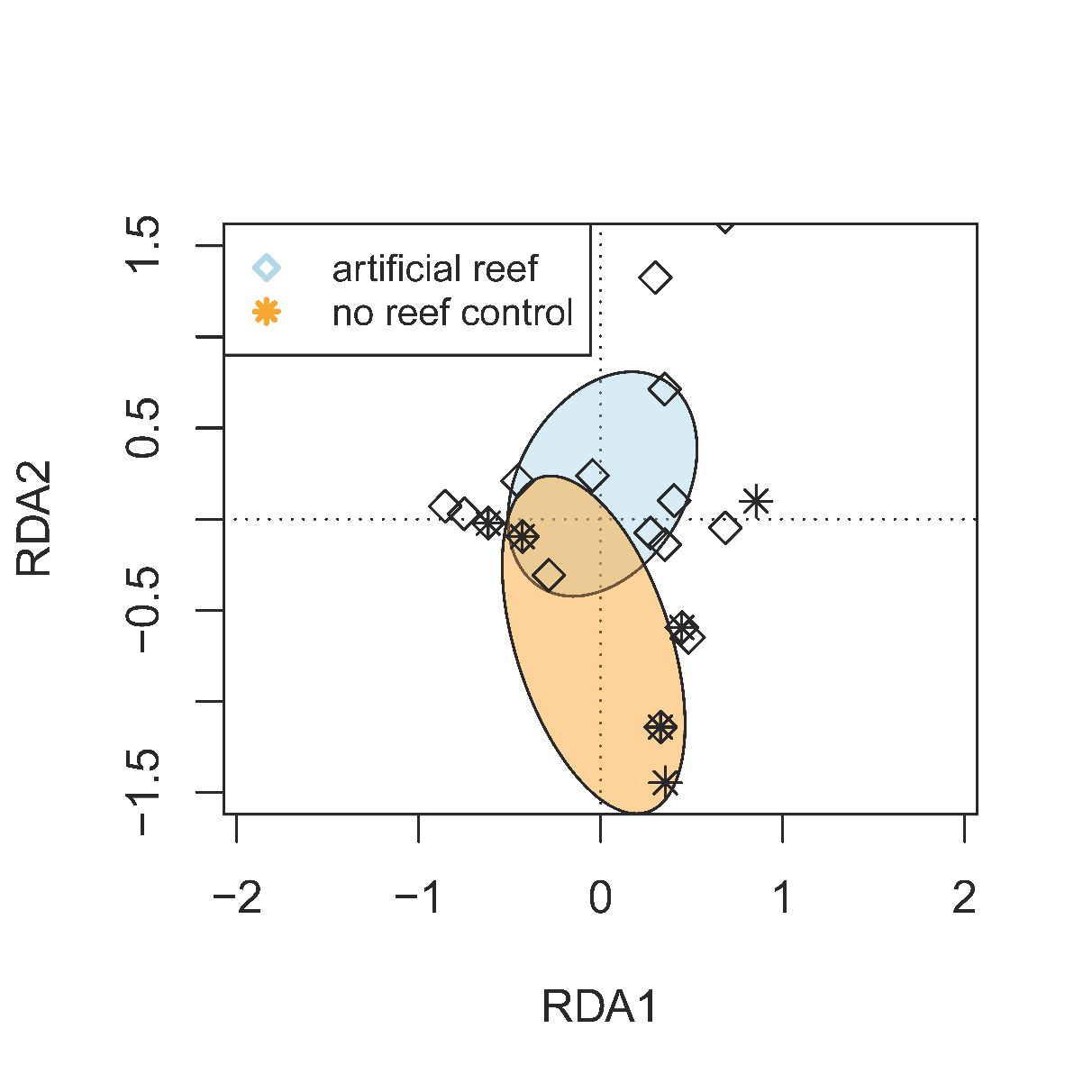


Figure S2:2. RDA plot showing sample scores and the separation of the cage reef treatments (reef cage or not) along RDA axis 2. Diamonds show the sample scores for plots with an artificial reef cage (artificial reef), the star symbol show the sample scores for plots without any added reef cages (no-reef control). The blue ellipse show the standard deviation around the centroid (mean) for the artificial reefs, the orange ellipse show the standard deviation around the centroid (mean) for the no-reef control.


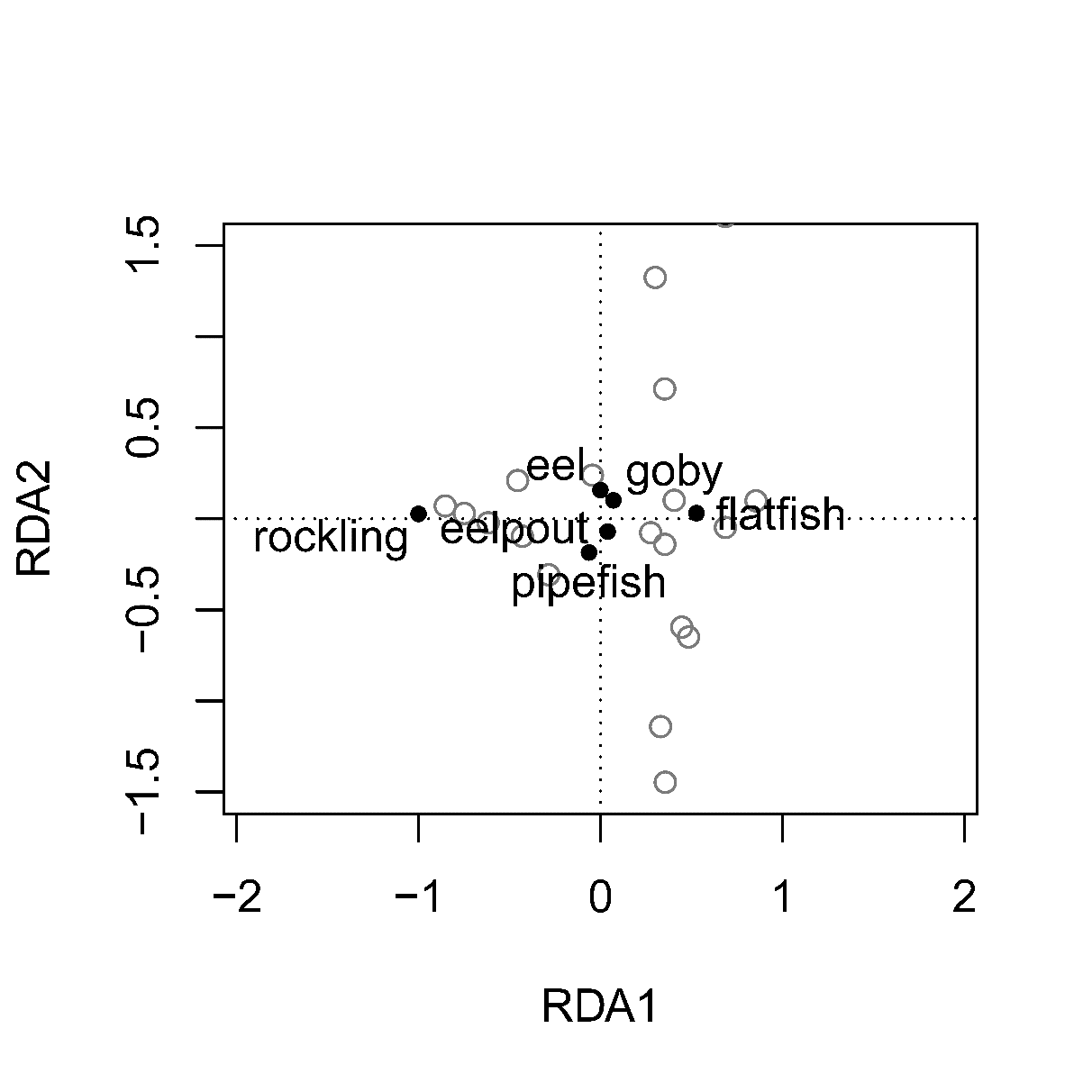


Figure S2:3. RDA plot showing the position of sample (grey circles) and species scores (black dots) along two ordination axes constrained by season and reef cage treament (reef-cage or not). Only species that were significantly explained by the RDA axes 1 and 2 were included in the plot.


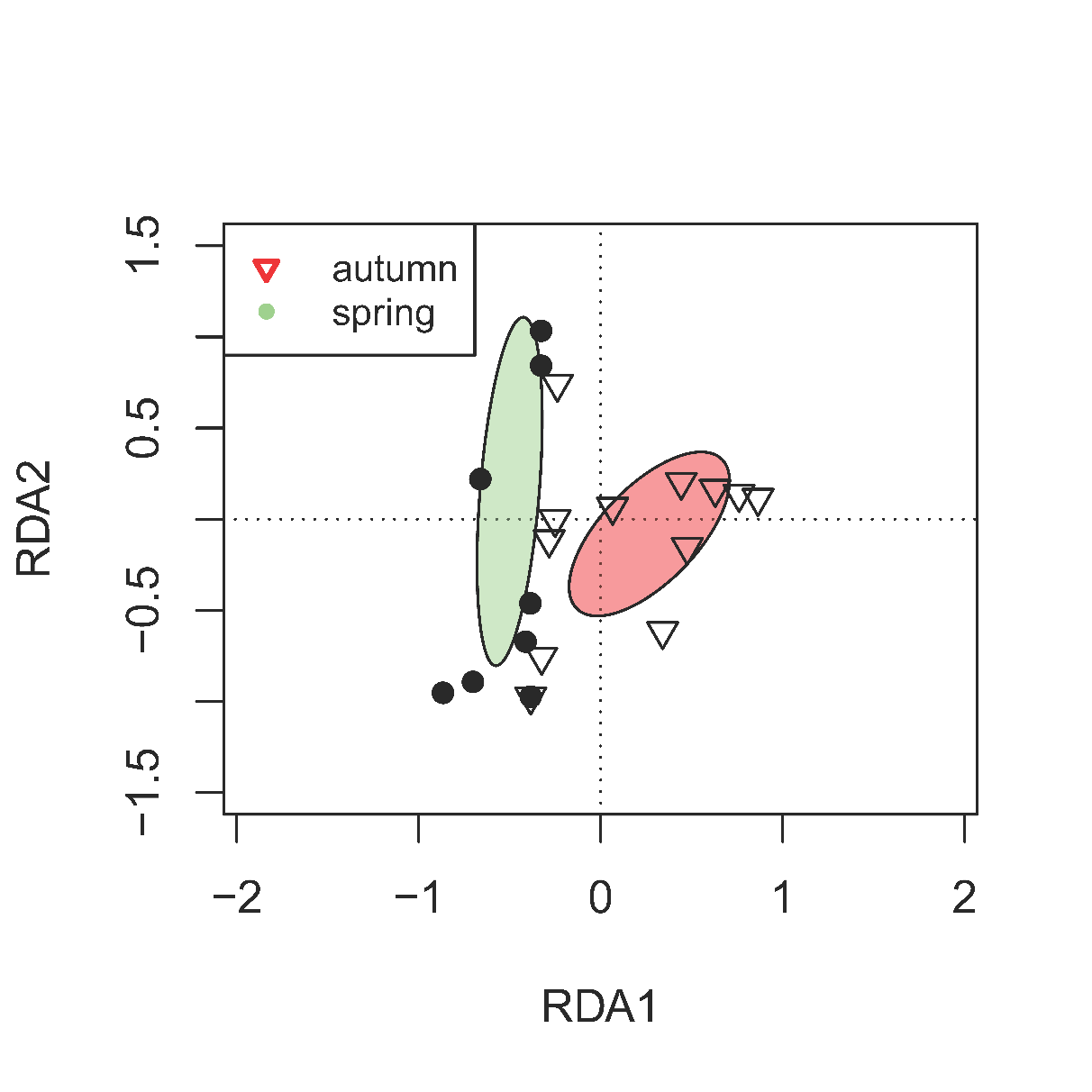


Figure S2:4. RDA plot showing sample scores and the separation of season along RDA axis 1. Triangles show the sample scores for autumn and black dots show the sample scores for spring. The red ellipse show the standard deviation around the centroid (mean) for autumn, the green ellipse show the standard deviation around the centroid (mean) for spring.


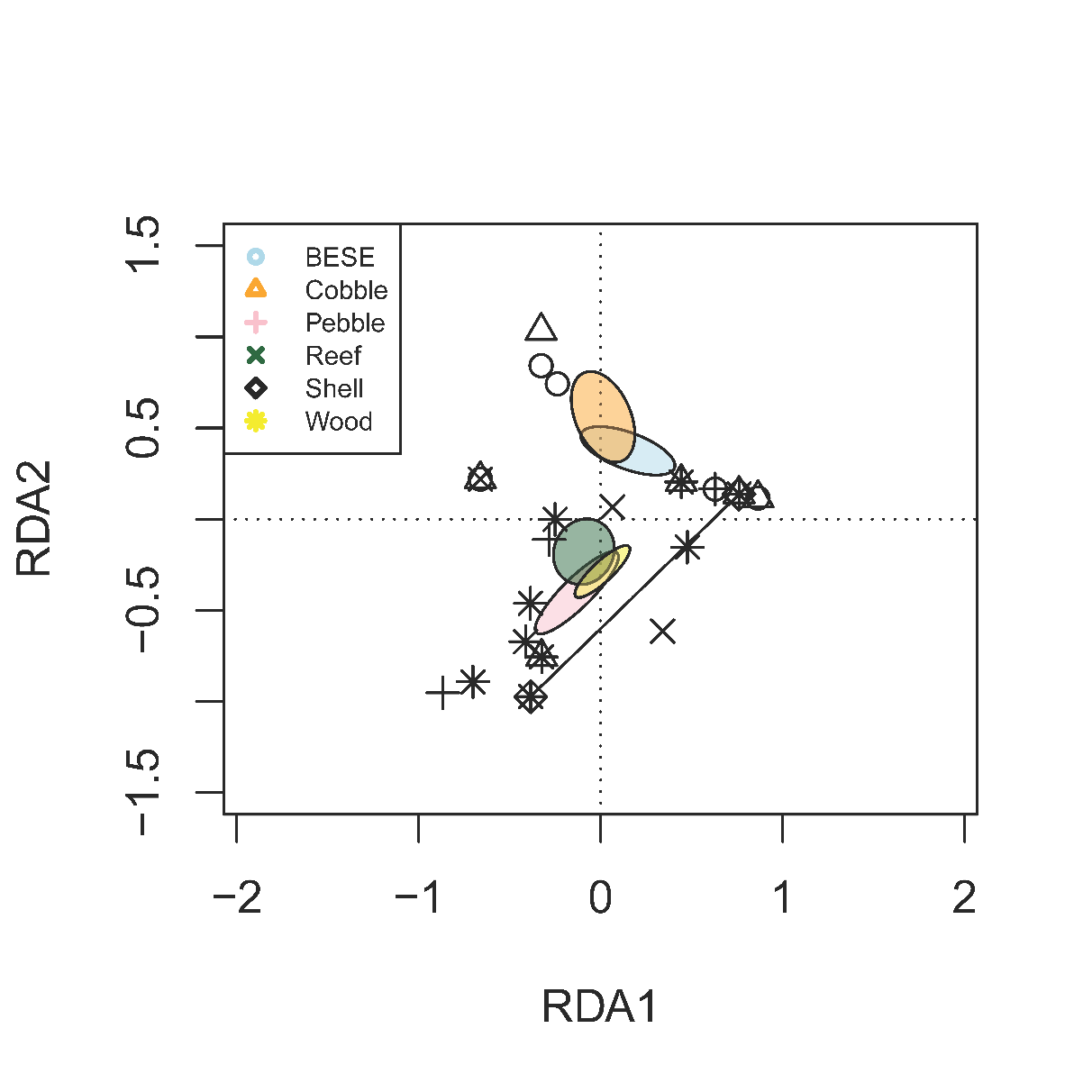


Figure S2:5. RDA plot showing sample scores and the separation of the type of cages along RDA axis 2. Blue circles show the sample scores for cages with BESE elements; orange triangles for cages with cobbles; pink plusses for cages with pebbles; green x’s for cages with boulders coasted with cacerous material; black diamonds for cages with empty cockle shells; and yellow stars for cages with wood. The ellipses show the standard deviation around the centroid (mean) for each artificial reef treatment.


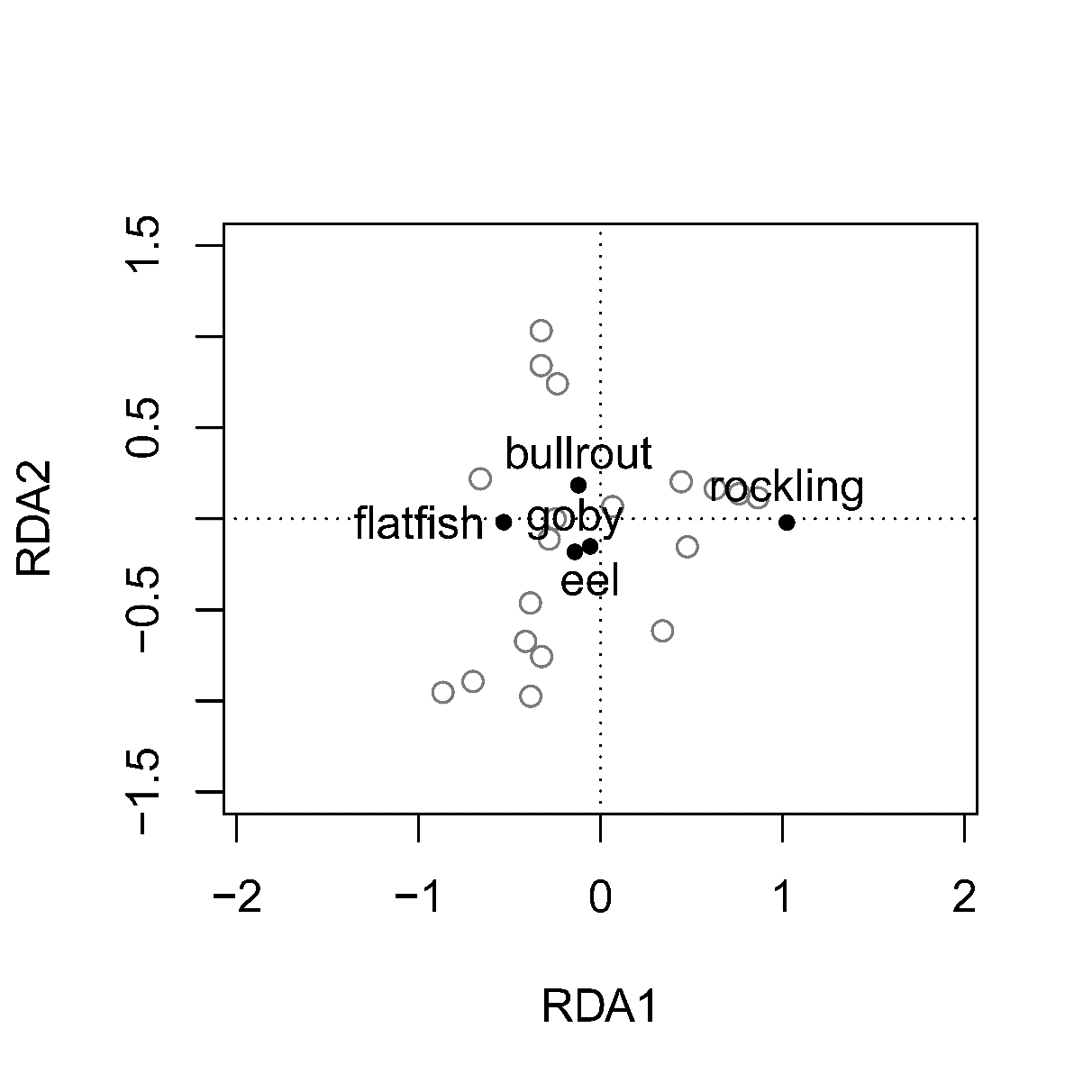


Figure S2:6. RDA plot showing the position of sample (grey circles) and species scores (black dots) along two ordination axes constrained by season and type of reef cage (6 levels: BESE elements; cobbles; pebbles; boulders; cockle shells; and wood.). Only species that were significantly explained by the RDA axes 1 and 2 were included in the plot.
